# Feasibility of mHealth Strategies to Promote Use of Wrist-Worn Alcohol Biosensors Among Young Adults With HIV: Protocol for the Engage Microrandomized Trial

**DOI:** 10.2196/69966

**Published:** 2025-11-12

**Authors:** Stephanie M Carpenter, Stuart Case, Mia Liza A Lustria, Laura Reid Marks, Qinggang Yu, Karen MacDonell, Sylvie Naar, Yan Wang

**Affiliations:** 1 College of Health Solutions Arizona State University Phoenix, AZ United States; 2 Department of Health Policy and Management, School of Public Health University of Pittsburgh Pittsburgh, PA United States; 3 School of Information, College of Communication and Information Florida State University Tallahassee, FL United States; 4 Center for Translational Behavioral Science, Behavioral Sciences and Social Medicine, College of Medicine Florida State University Tallahassee, FL United States; 5 Survey Research Center Institute for Social Research University of Michigan–Ann Arbor Ann Arbor, MI United States; 6 Department of Epidemiology, College of Public Health and Health Professions and College of Medicine University of Florida Gainesville, FL United States

**Keywords:** HIV, microrandomized trial, transdermal alcohol sensor, mobile health, wearable technology, self-monitoring, gamification, engagement

## Abstract

**Background:**

Alcohol use among young adults with HIV is disproportionately high compared to other age groups with HIV, despite its negative health-related consequences. However, alcohol use interventions tailored to the interests and needs of young adults with HIV are scarce. Self-management interventions that include self-monitoring components have the potential to improve chronic illness outcomes and mitigate alcohol misuse. Although wearable technologies promote self-monitoring behaviors and facilitate the delivery of personalized feedback, people rarely adhere to the long-term use of wearables.

**Objective:**

The primary aim of this pilot study is to test the feasibility and acceptability of translating theoretically grounded reciprocity and personalized feedback strategies. These strategies will be delivered through app-based messages to promote engagement in wearing a wrist-worn alcohol biosensor among young adults with HIV.

**Methods:**

A total of 40 young adults with HIV (aged 18-29 years) will be enrolled in a 30-day pilot microrandomized trial (MRT). The MRT is an experimental design that facilitates the development and optimization of mobile health just-in-time adaptive interventions. Participants will wear the BACtrack Skyn biosensor, a wrist-worn transdermal alcohol biosensor, and play a smartphone-based game geared toward earning points to care for virtual animals. Longer alcohol biosensor wear time will translate into earning more game points. Every morning and evening, participants will be randomized at equal probability to receive (1) a reciprocity message offering a no-strings-attached gift of either 100 game points or US $1, (2) a message with personalized feedback about their Skyn biosensor wear time, or (3) no message.

**Results:**

Data collected during this 30-day MRT will be used to examine the feasibility and acceptability of translating theoretically grounded strategies into messages suitable for a mobile health alcohol intervention tailored to young adults with HIV. Recruitment for this study began in spring 2024, with data collection wrapping up in spring 2025.

**Conclusions:**

This pilot MRT will provide valuable feasibility and acceptability data and set the stage for a full-scale MRT to optimize the integration of reciprocity and personalized feedback into a just-in-time adaptive intervention that increases engagement in biosensor-based alcohol self-management among young adults with HIV.

**Trial Registration:**

Clinicaltrials.gov NCT05431855; https://clinicaltrials.gov/study/NCT05431855

**International Registered Report Identifier (IRRID):**

DERR1-10.2196/69966

## Introduction

### Background

High-risk alcohol consumption, defined as drinking ≥4 standard drinks for women and ≥5 drinks for men in 1 sitting, is a major health concern among young adults aged between 18 and 29 years [1-3]. Alcohol consumption is especially common among young adults with HIV. Research shows that young adults with HIV exhibit disproportionately high rates of alcohol use disorder (AUD) compared to the general youth population, as well as other age groups of people with HIV [4]. High-risk alcohol consumption in this population can lead to detrimental health-related consequences, such as a higher likelihood of HIV transmission through risky sexual behaviors, increased risks for comorbidities such as declined liver and cognitive functioning, and lower adherence to antiretroviral medications that may lead to viral nonsuppression and disease progression [5-9].

Despite the clear need for interventions to address high-risk alcohol consumption among young adults with HIV, few interventions are tailored to meet their needs or interests. Existing alcohol interventions for young adults with HIV often fail to sustain behavior change beyond the intervention period, limiting their long-term effectiveness [10-17]. Furthermore, young adults with HIV may fear stigma [18] and often have limited access to intensive, in-person interventions; clinical resources [19-21]; and transportation [22,23]. Mobile health (mHealth) interventions delivered through smartphones and wearable technologies are promising for young adults with HIV because 98% of US young adults own smartphones [24]. However, there are a limited number of real-time mHealth alcohol use interventions for young adults with HIV. One promising approach to improving chronic illness outcomes and mitigating high-risk alcohol use and AUD is through the use of mHealth interventions to deliver self-management strategies such as self-monitoring.

Self-monitoring is a key strategy for supporting behavior modification across a number of health interventions, ranging from those focused on increasing weight loss and physical activity to reducing substance use, including unhealthy drinking habits [25-28]. Self-monitoring allows individuals to monitor their behaviors (eg, alcohol consumption); assess progress on their behavioral goals; and, through self-reflection and reinforcement, adjust their behaviors to achieve their goals [27-29]. Such self-monitoring behavior is a key component of many successful behavior change interventions [30].

Wearable technologies support systematic and continuous self-monitoring and the collection of individual data that can be used to generate personalized feedback and other information to support self-monitoring. However, the benefits of wearables for self-monitoring can only be achieved if used consistently [31]. This presents a challenge because many people abandon wearable technologies after only minimal use [32,33]. These findings suggest the need for research to identify different strategies for increasing consistent engagement with mobile-based, self-monitoring technologies [34,35].

Several theoretically grounded engagement strategies have been shown to help promote behaviors that improve self-monitoring [3,33]. Providing personalized feedback is a simple and effective way to motivate individuals to self-monitor their behaviors, including alcohol consumption [30]. For instance, in a meta-analytic review of randomized controlled trials testing alcohol interventions, Scott-Sheldon et al [36] found that personalized feedback was a significant predictor of a variety of alcohol-related outcomes, including quantity of alcohol consumed, frequency of heavy drinking, and alcohol-related problems. Reciprocity, another theoretically grounded strategy, can also promote engagement in behaviors that enhance self-monitoring. Reciprocity refers to an innate tendency of humans to want to return favors and acts of kindness [37-39]. For instance, if an individual receives a small amount of money as an incentive, regardless of whether or not they engage with (eg, use) an intervention, this may inspire them to reciprocate or give back by increasing their investment in the intervention, thus promoting greater self-monitoring of their drinking behaviors.

Self-monitoring can be further enhanced by incorporating those engagement strategies into a just-in-time adaptive intervention (JITAI). JITAIs aim to provide real-time mHealth support in a person’s natural setting. They adapt over time to changes in an individual’s internal states and surrounding contexts to deliver the right type of support at the right time [40-42]. For instance, a JITAI can be designed to deliver the right type of message (eg, either reciprocity or personalized feedback) at the right time (eg, first thing in the morning before a busy day when the person is more receptive and thus willing and able to use an intervention message) to engage participants in behaviors such as self-monitoring [41].

A microrandomized trial (MRT) is a novel experimental study design that helps optimize (ie, construct) a JITAI. During an MRT, participants are randomized repeatedly (eg, several times per day) over time (eg, several days or weeks) to different intervention options (eg, messages using reciprocity vs personalized feedback). The data collected from an MRT can be used to analyze which intervention option is more effective in increasing the target behavior (eg, self-monitoring) and at what time, under what contexts, or to whom it is more effective. This information can in turn be used to develop a JITAI that delivers the right type of support at the right time to promote engagement in self-monitoring behaviors [43,44].

### Objectives

This study is a first step toward building a JITAI to improve the engagement of young adults with HIV through increasing wear time of the BACtrack Skyn, a wrist-worn alcohol biosensor [45]. Over the past 5 years, wrist-worn transdermal alcohol biosensors have emerged as a promising new tool for continuous monitoring of alcohol use (for more detailed review on wrist alcohol biosensors, refer to the studies by Wang et al [46-48]). These alcohol biosensors can detect alcohol use passively by measuring the small fraction (<1%) of ingested alcohol that is excreted through the skin and producing continuous transdermal alcohol concentration [49] readings every 20 seconds that reflect near real-time alcohol consumption. Although the new-generation of wrist-worn alcohol biosensors are much more user-friendly compared to the previous generation of transdermal alcohol biosensors (eg, the SCRAM continuous alcohol monitor anklet; AMS Inc), adherence to wearing the sensor is still a challenge, especially for long-term use that is essential for sustaining self-monitoring and improving health outcomes [50].

The primary aim of the proposed pilot study is to test the feasibility and acceptability of integrating theoretically grounded reciprocity and personalized feedback engagement strategies into an mHealth intervention designed to increase engagement in Skyn biosensor wear time among young adults with HIV. A secondary aim is to explore for whom and under what conditions different strategies are best for promoting the engagement (Skyn biosensor wear time) of young adults with HIV.

## Methods

### Population

Up to 40 young adults with HIV (aged 18-29 years) will be enrolled to participate in this study. We expect an attrition rate of approximately 25%, yielding an anticipated final sample size of 30 participants. The HIV status of young adults with HIV will be verified in one of the following three ways: (1) viral load results obtained from a health care professional (eg, primary care physician) that is uploaded directly by participants, (2) viral load results uploaded by the provider upon request, or (3) completion of a dried blood spot test collection kit sent to the participant.

### Recruitment and Screening

Participants will be recruited through social media platforms (eg, Instagram) via a recruitment service (ie, Build Clinical) and from HIV clinics across the state of Florida. Interested individuals will be screened based on the following eligibility criteria: participants must be aged between 18 years 0 months and 29 years 11 months; reside in the state of Florida for the duration of their study enrollment; have had at least 1 alcoholic beverage in the last 30 days; have been diagnosed with HIV; not be pregnant at the time of screening; have internet access via smartphone, tablet, or computer; and be able to read and understand English. Participants must also be willing and able to participate in the study for the entire 30-day study period. Participants will be informed that they can earn up to US $240 for completing all portions of the study. Participant recruitment began in spring 2024.

### Data Collection Procedures

Eligible participants will be instructed to watch a web-based consent video and read an informed consent document outlining the details of the study, including its risks and benefits. Participants who consent to participate will complete a web-based survey to collect sociodemographic information (eg, race, ethnicity, gender, and socioeconomic status) and baseline data on individual characteristics. The individual characteristic items include measures of risk taking, depression, alcohol use, substance co-use (eg, tobacco, nicotine, prescription medications, and opioids), perceived stress, and personality. All study staff interacting with participants are trained in crisis mitigation and follow a comprehensive protocol to evaluate levels of distress risk and corresponding risk management. Any participant identified as high risk based on their responses to baseline questionnaires will be directly referred to a licensed clinical psychologist on the study team for further assistance. Once participants have completed the baseline survey, they will be scheduled for a videoconference-based onboarding session with research staff using Zoom (Zoom Video Communications, Inc).

Before the onboarding session, participants will be mailed a BACtrack Skyn biosensor to a preferred address, along with instructions for how to download and use each study-related app. All participants who do not own an iPhone will also be mailed a study iPhone that is compatible with the Skyn sensor (which is currently only compatible with an iOS system) to facilitate study inclusiveness and data collection. All study-related apps will be preinstalled on the study iPhone.

The onboarding session will take place virtually over Zoom. During this session, research staff will train participants on how to use the Skyn biosensor and guide them on how to download (if using their own iPhone) and use its associated app (Skyn app), an ecological momentary assessment (EMA) app for momentary and daily brief surveys on alcohol use, and an engagement wrapper (e-wrapper) app (described in the e-Wrapper App section). Participants will also receive instructions on how to sync or upload their Skyn sensor data using the Skyn app. Participants will be informed that they may reach out to study staff should they have questions or issues related to the study materials.

### Study Design

Participants who complete all baseline measures will be enrolled in a 30-day pilot MRT study (refer to the MRT Design section). Participants will be instructed to wear the Skyn biosensor for as many hours a day as possible during the 30-day study period. The research staff will make it clear that the sensor should be worn continuously and can only be removed for the purpose of charging the battery or when it is likely to be submerged under water (eg, baths and swimming) to avoid potential damage to the sensor. The battery life of the Skyn sensor is approximately 10 to 14 days, and participants will be instructed to charge the device ideally when they will not be wearing it anyway (eg, while taking a bath).

Each morning, participants will receive a short EMA survey on their smartphone to self-report their drinking and other substance use behavior in the past 24 hours, as well as their adherence to antiretroviral therapy, sexual behaviors, quality of sleep, and any difficulties they encountered with the use of the Skyn biosensor.

In addition to the morning EMAs, participants will also receive random EMAs 3 times a day to assess their current mood, stress, urge to consume alcohol, confidence and self-efficacy to resist drinking, current location, and alcohol and other substance use behaviors since the time of the last EMA. Individuals who self-report drinking alcohol will initiate an additional event-contingent survey that gathers more details about the location where they consumed alcohol, the type of alcoholic beverage they consumed (eg, beer or liquor), and whether they were in the presence of other people.

### e-Wrapper App

Wearing the Skyn sensor increases one’s awareness of their drinking behaviors and will serve as a tool to promote the self-monitoring of alcohol use. Thus, it is critical that participants wear the sensor regularly throughout the intervention period. As participants in mHealth studies frequently abandon the use of wearable devices [31], it is necessary to identify strategies to enhance engagement [3,33,51], specifically time spent wearing the Skyn sensor. Principles of computer-human interaction suggest that incorporating gamification into an intervention can promote engagement [52,53]. Gamification [54,55] integrates game-like design components and principles into interventions to provide meaningful and sophisticated techniques to engage participants in mHealth behavior change interventions [56-58]. However, relying on incentives (eg, money and game points) alone for reward can undermine intrinsic motivation if an individual does not feel a personal connection to the activity [3,57,59,60], in this case, to the game.

To leverage the positive impacts of gamification strategies and promote greater intrinsic motivation through building a connection to the intervention, we developed an e-wrapper app. mHealth intervention apps tend to focus on the behavioral (eg, self-monitoring) tasks at hand, for instance, providing people with a platform for listing their goals or tracking when they drink alcohol. The e-wrapper app described here is novel because it can be paired with (eg, “wrap around”) existing apps that aid in self-monitoring activities to offer additional incentives that can elicit interest and connection to the intervention, further enhancing intrinsic motivation to engage individuals in the self-monitoring activity. A variety of gamified apps, including those with virtual characters such as avatars and animals, have been shown to promote engagement in young adult populations [52,61-63], including young adults with HIV [64-69]. The gamified e-wrapper app is designed to sustain intrinsic motivation by providing an enjoyable interactive experience where the e-wrapper game challenges participants to make choices about caring for virtual pets. Upon starting the game, participants are initially given a virtual pet (eg, giraffe) living in a preselected “habitat” and 200 points to use toward care of the pet.

**Figure 1:**
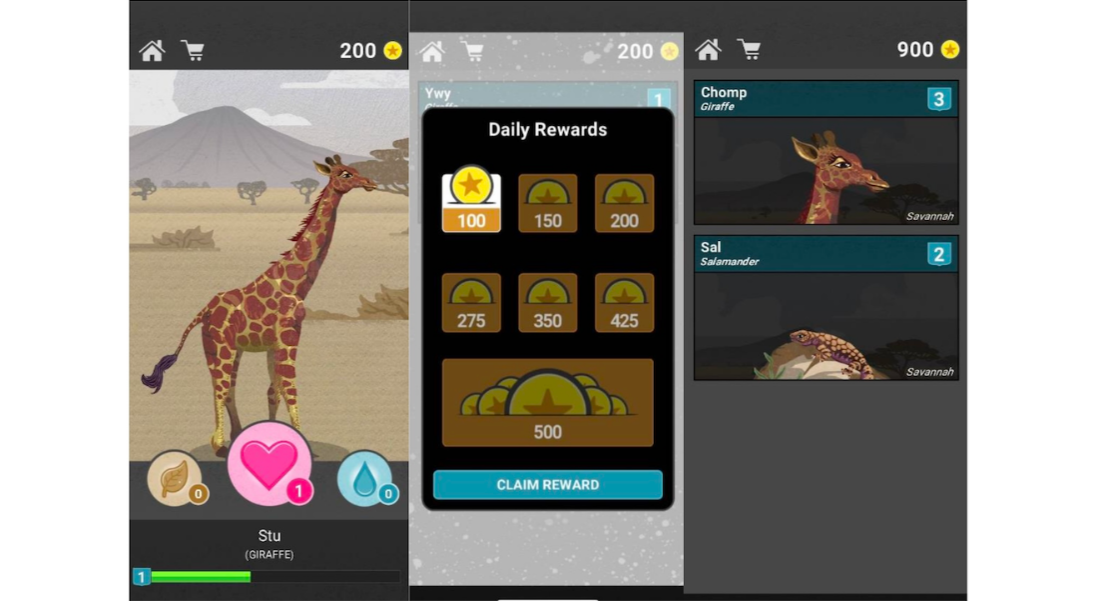
Gamified engagement wrapper (e-wrapper) app used to further promote engagement in the self-monitoring intervention.

In addition to their first virtual pet, participants may also purchase additional pets from a list of options: a giraffe, an ostrich, or a salamander. Participants can also purchase a virtual baby pet (or in the case of the ostrich, an egg) with the goal of raising the pet into adulthood. Several reward-based engagement strategies are embedded within this gamified app (Figure 1) to further promote engagement, but these stay consistent across the study for all participants and are thus not part of the microrandomization. For instance, participants can personalize each pet (eg, giving names) to increase their connection with their virtual pets. As part of the game, participants must care for each of their pets by feeding them, providing water, and offering treats. They can use points to purchase 1-time quantities of food and water, meal packages that cover 5 feedings, individual treats or treat bundles, additional habitats (eg, forest), and various hats to dress up their pets (refer to Table 1 for full point allocations).

Participants can also earn additional points to care for their pet or pets by opening the app on consecutive days, by wearing their Skyn biosensor for a longer amount of time (Table 1), and through an additional engagement message strategy described in the MRT Design section. This metric for points based on wear time (engagement) provides the highest reward to those who wear their sensor at least 80% of the time and also allows participants to remove the Skyn biosensor when showering or charging the device.

**Table 1:** e-Wrapper ranch game point allocation.

Game costs		Game points award schedule					
Items to purchase	Point totals	Consecutive day e-wrapper app opening (each week)	Point totals	Skyn biosensor wear time (h/d)	Point totals	Point-based reciprocity messages	Point totals
Food	50	Day 1	100	0	0	Each message	100
Water	50	Day 2	150	1-18	100	—^a^	—
Meal packages (food or water for 5 feedings)	200	Day 3	200	19-24	200	—	—
Individual treats	150	Day 4	275	—	—	—	—
Treat packages (5 treats)	600	Day 5	350	—	—	—	—
Accessories (eg, hats)	500-4000	Day 6	425	—	—	—	—
Habitats	2000	Day 7	500	—	—	—	—
Animals	1000	—	—	—	—	—	—

^a^Not available.

### MRT Design

#### Overview

An MRT is an experimental design that provides empirical data to facilitate the development and optimization of mHealth JITAIs [40,43,44]. MRTs accomplish this by providing information on the proximal effects of specific interventions, changes in these effects over time, and the psychological and contextual factors that moderate these time-varying effects [43,44]. Here, participants are randomized repeatedly at equal probability to receive message prompts framed around reciprocity, personalized feedback, or to receive no message (refer to the Message Strategies section). Rerandomizing to different options throughout the study allows for the collection of data on under what internal states and external contexts, as well as at what time in the intervention [3], different intervention options may be more or less effective. Findings from an MRT allow researchers to better determine how and when an intervention component should be delivered to optimize the efficacy of that intervention. JITAIs have become increasingly common in recent years for preventing and treating substance use disorders [3,40], as JITAIs adapt to the changing internal and external contexts of individuals to provide support in the moments when an individual is most in need [40-42]. For example, a JITAI can promote engagement by intervening in those moments when an individual is likely to abandon use of the Skyn biosensor. This pilot MRT study will serve as the foundation for the development of a future JITAI.

#### Message Strategies

To promote greater engagement with the BACtrack Skyn biosensor, we selected two theoretically grounded strategies that may be effectively translated into messages suitable for delivery through the e-wrapper mHealth app: (1) reciprocity and (2) personalized feedback.

#### Reciprocity

As described briefly in the Introduction section, reciprocity relies on the notion that giving a small, no-strings-attached reward will activate a human tendency to return favors and kind actions [37-39]. In other words, a small gift that is not contingent on the target behavior (Skyn biosensor wear time) will plausibly inspire the person to reciprocate by doing the desired behavior (wearing the Skyn biosensor). Prior research has shown that reciprocity can be translated into messages suitable for an mHealth intervention [33,40], making this strategy particularly appealing. The ideal form of the gift, however, remains unclear. Here, we operationalize reciprocity as either a gift of 100 points to use for care of the virtual pet in the e-wrapper app or as a US $1 gift. Points will be awarded in the e-wrapper app immediately for use in the game, while free money gifts will be paid to participants at the end of the study. We anticipate each participant may be awarded approximately 1000 points and US $10 total across the course of the study. Providing 2 different types of reward will help us determine if and when providing free game points or money is more effective for increasing the Skyn biosensor wear time.

#### Personalized Feedback

A second strategy that can promote Skyn biosensor engagement is personalized feedback about wear time. Personalized feedback entails directly showing an individual how much they are engaging in a specific behavior. This feedback enhances participants' awareness of their behavior and offers a foundation to guide future improvements [15]. By delivering encouraging messages that highlight how much time people are spending wearing the Skyn biosensor, it is possible to increase one’s motivation to wear the biosensor for a greater duration of time.

#### Message Development

The study team translated the reciprocity and personalized feedback strategies into initial messages suitable for delivery in an mHealth intervention. To do this, we obtained feedback on these messages from community advisers in two phases. First, we collected feedback through a survey of Amazon Mechanical Turk workers consisting of members of the general population who were recruited using the CloudResearch Platform [70]. Next, we interviewed members of an expert advisory board consisting of individuals with a similar sociodemographic makeup (eg, young adults, majority from a racial and ethnic minority group) as the target population. Community advisers in these formative stages rated the messages in terms of how much they liked the messages and how likely they thought the messages would be able to promote the intended behavior (eg, wearing the Skyn sensor). They also provided suggestions for how to improve the messages. This feedback was used to iteratively refine the messages in preparation for the pilot MRT study. Messages were designed so that clicking on the message would automatically open the e-wrapper app. Table 2 includes examples of these messages.

**Table 2:** Sample engagement messages generated by the study team and revised by community advisers.

Message type	Message
Reciprocity (money)	“Got the blues today? How about some green! (app)’s added a $1 gift to your (cash account).”
Reciprocity (game points)	“Hey there! Your animal(s) miss you. Here are 100 free bonus credits from (app) to your Ranch account. Treat them today!”
Personalized feedback	“Look at you being productive! You wore your Skyn sensor for <<XX>> hours yesterday. Keep going!”

#### MRT Randomization

**Figure 2:**
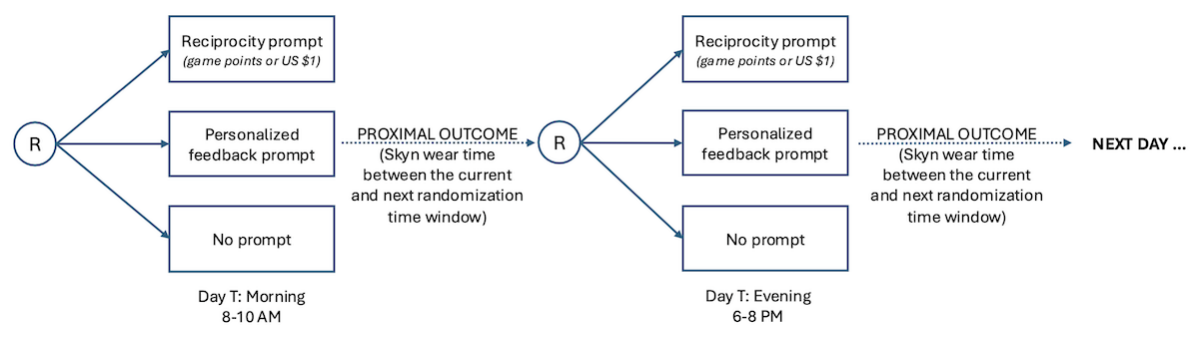
Microrandomized trial design. Microrandomizations occur twice daily for 30 days.

A key feature of the MRT design is that participants are randomized at various points in time (ie, decision points) across the study to answer scientific questions about if and when the delivery of specific types of messages (ie, reciprocity or personalized feedback) is likely to promote an outcome of interest [33,40,41,51], such as engagement. In this pilot study (Figure 2), during 2-hour time windows every morning (between 8 and 10 AM) and evening (between 6 and 8 PM) for 30 days, participants will be randomized at equal probability to receive (1) a reciprocity message, (2) a personalized feedback message about their Skyn biosensor wear time, or (3) no message. When the reciprocity messages are delivered, there is an equal probability they contain either a no-strings-attached gift of (1) 100 game points or (2) US $1. Participants will not receive any messages one-third of the time to explore whether, under some internal or external conditions, it may be best to not send a message. Messages will be sent as push notifications from the e-wrapper app to the study iPhones.

### Poststudy Assessment

At the end of the 30-day MRT phase, participants will be asked to complete a poststudy assessment online. In this assessment, they will self-report on the feasibility and acceptability of integrating the theoretically grounded strategies into messages to promote engagement in Skyn biosensor wear time. Participants will also provide ratings of feasibility and acceptability (including liking and usability) regarding use of the Skyn biosensor and e-wrapper app.

### Study Compensation

The total compensation that participants can earn for completion of all aspects of the study is US $240. Table 3 outlines the compensation schedule, including compensation type and amount earned for completing each component of the study. For the EMA bonus, if participants complete ≥80% of delivered EMAs, then they will receive a US $10, US $20, US $30, and US $40 bonus for each of the 4 weeks. Note that while recruitment began in spring 2024, the bonus was implemented after the first 4 participants were recruited to improve study retention. If participants fall below 80% compliance, then they will restart at the week 1 bonus rate of US $10. Including the base pay and bonuses, participants can receive up to US $160 for EMA completion. Finally, US $100 will be deducted from total earnings if the study iPhone and BACtrack Skyn biosensor are returned damaged or >2 weeks from the time of study completion, and no compensation will be provided if devices are never returned. Research staff will monitor data collection immediately following onboarding of each participant and contact them via phone (call or SMS text message) and email as soon as possible if data are missing on the EMAs, Skyn biosensor, or e-wrapper app within the first 2 days. We will similarly follow up with participants who are noncompliant with the study protocol, defined as missing data on the EMAs, Skyn biosensor, or e-wrapper app for more than a couple of days, or have not returned the equipment.

**Table 3:** Study compensation.

Compensation type			Amount (US $)
Baseline survey			25
HIV viral load DBS^a^ sample			10
DBS sample for phosphatidylethanol test			10
**EMA^b^ completion rate (%)**			
	≥80	60	
	60-79	50	
	50-59	40	
	40-49	30	
	20-39	20	
	0-20	10	
**EMA bonus schedule (for streaks of ≥80% completion)**			
	Week 1	10	
	Week 2	20	
	Week 3	30	
	Week 4	40	
Poststudy follow-up survey			35
Maximum possible			240

^a^DBS: dried blood spot.

^b^EMA: ecological momentary assessment.

### Statistical Considerations and Data Analysis

#### Study Outcomes

The primary purpose of this pilot MRT study is to assess the feasibility and acceptability of two key aspects of the intervention: (1) delivering messages to promote engagement in wearing the Skyn biosensor and (2) linking engagement (ie, Skyn wear time) to playing the e-wrapper game. In addition, we will evaluate whether delivering a message prompt versus no message prompt will increase Skyn biosensor wear time between the current and the next randomization time window (eg, the 8 h between the morning and evening randomization windows; Figure 2).

#### Feasibility and Acceptability Measures

As the main purpose of a pilot MRT is to assess the feasibility and acceptability of the study to refine the procedures and materials in preparation for a full-scale MRT, participants will be asked to complete a post-MRT assessment with questions related to how much they liked the messages and how much they thought the messages encouraged them to engage. For instance, participants will be asked to rate “How much did you like the messages you received?” on a scale ranging from 1 (*not at all)* to 5 (*very much*). Information will also be gathered on participant perceptions of the number and frequency of messages received. Participants will then rate the feasibility and acceptability of using the e-wrapper app and indicate how much they liked the app and its usability. Metrics of feasibility will also be assessed from paradata, including information on how often message notifications were clicked, how often the app was opened, how much time participants spent using the app, and how long participants wore the Skyn biosensor.

#### Analysis Plan

Feasibility and acceptability data will be analyzed using descriptive statistics (eg, frequencies, means, medians, and proportions). Results of this pilot MRT study will inform the development of a future full-scale MRT to test the optimal timing for delivering reciprocity and personalized feedback messages to promote proximal (time between the current and next randomization) sensor wear time (our proxy for self-monitoring). The pilot MRT data will be analyzed using a novel approach specifically developed to ensure unbiased estimates of causal effects of time-varying treatments for binary outcomes [71]. These analyses will pool time-varying, longitudinal data across all study participants and allow for marginal comparisons, that is, comparisons of intervention options without conditioning on participants’ full history of prior treatment [71]. Such marginal comparisons allow us to answer questions about the causal relationship between delivering a message and the outcome of interest (ie, Skyn biosensor wear time), as well as how such effects differ based on an individual’s internal states and external contexts. This analysis approach is preferred to other common methods for analyzing MRT data, such as random-effects models and generalized estimating equations, which may result in biased causal effect estimates for time-varying treatments and covariates [71]. This MRT will investigate whether providing any engagement strategy (either reciprocity or personalized feedback) will affect sensor wear time following microrandomization. Specifically, we will test whether there is an average proximal effect of the engagement strategies on the amount of sensor wear time in the window between the current and next randomization at each decision point during the study. Our proximal outcome is 75% engagement, which is a common average engagement goal for mHealth modalities, including those involving wearables [72-75]. For the pilot analysis, wear times of ≥75% of the total time between the current and next randomization (eg, ≥6 h when there are 8 h between prompt microrandomizations) will be coded as 1=engaged and wear times <75% (eg, <6 h when there are 8 h between prompt microrandomizations) will be coded as 0=not engaged. This will provide flexibility by considering instances when participants may not wear the sensor, for example, when they are bathing or charging the Skyn biosensor.

#### Power Analysis and Sample Size

Although the main purpose of this pilot study is to assess feasibility and acceptability, we conducted preliminary power analyses to begin exploring the comparison of receiving any prompt versus no prompt on Skyn biosensor wear time. Across the 30 days of the pilot MRT study, participants will be randomized twice daily with one-third probability to receive (1) reciprocity prompts (US $1 or 100 e-wrapper game points), (2) personalized feedback prompts (indication of the percentage of time they have worn the Skyn sensor either in the past day or over the course of the study), or (3) no prompts. For this exploratory study, a sample size of 40 participants will enable us to detect an average proximal treatment effect on a relative risk scale of at least 1.32 (ie, 16% improvement with any prompt, assuming participants will wear the sensor ≥75% of the time between prompt microrandomizations at least 50% of the total time) in favor of receiving any reciprocity or personalized feedback prompt (vs no prompt), with 80% power. These calculations are based on a 2-sided 0.05 type I error rate, 25% attrition, and 25% anticipated availability (ie, to receive a message; for instance, are not busy at work, commuting, or did not turn off SMS text message notifications on their phone) and were conducted using an established MRT power analysis calculator [76]. The conservative attrition and availability rates were identified based on existing work suggesting that populations with chronic diseases such as HIV have generally high attrition and low availability in mHealth-based research [41]. We aim to recruit 40 participants, anticipating that 30 will complete the trial, accounting for an expected 25% attrition rate.

### Ethical Considerations

This pilot MRT is registered with Clinicaltrials.gov (NCT05431855) and approved by the Florida State University Institutional Review Board (STUDY00002534). Informed consent will be obtained from all individuals, and compensation disclosed, before enrollment in the study. Study IDs will be used to link data so no personal identifiers will be used in analyses. Once data linkage is completed, only deidentified datasets will be used for data analysis to protect participant privacy.

## Results

Recruitment for the study began in spring 2024, with data collection wrapping up in spring 2025. Analyses of the feasibility data and exploratory analyses of the pilot MRT prompt data will begin in summer 2025. Initial manuscript submissions for the feasibility and exploratory MRT findings are expected in fall 2025. We expect these manuscripts to be published in 2026.

## Discussion

### Anticipated Findings

We anticipate the main findings of the pilot study to indicate that intervention components are acceptable and study procedures are both acceptable and feasible. This study makes a key contribution to the existing literature by laying the initial groundwork to develop a future JITAI that uniquely combines theoretically grounded strategies and a gamified app to promote Skyn alcohol biosensor wear time among young adults with HIV. The main limitation of this study is that because it is a pilot, results will focus on feasibility and acceptability, and the trial is not powered to test the effects of different prompt types on Skyn alcohol biosensor wear time.

Feasibility and acceptability results from this pilot MRT study will guide refinements to study procedures and to intervention components to set the stage for a full-scale MRT. These results will also allow us to determine the percentage of missing data across study measures (eg, EMAs), which will guide the development of future retention strategies. Although the primary purpose of this study is to assess the feasibility and acceptability of the proposed procedures, data collected during this 30-day pilot MRT will also be used to begin exploring when and for whom a prompt may promote engagement (wear time) with the BACtrack Skyn alcohol biosensor. We also seek to understand how feasible and acceptable it is to connect Skyn biosensor wear time to the e-wrapper app, including how appealing participants find the app and how highly they rate it on likeability and usability. We plan to disseminate findings from this pilot study through manuscript publications, conference presentations, and community outreach (eg, newsletters).

### Conclusion

The future full-scale MRT will be appropriately powered to test the effects of different prompt types on Skyn biosensor wear time (engagement). Results of the full-scale MRT will optimize the integration of reciprocity and personalized feedback into a JITAI that provides the right support at the right time to increase engagement in biosensor-based alcohol self-management practices among young adults with HIV. By promoting greater Skyn biosensor wear time, this research has the potential to positively impact self-monitoring of alcohol use among young adults with HIV, which in turn may reduce high-intensity drinking behaviors and lower the risk of developing AUD.

## Abbreviations

AUD: alcohol use disorder
EMA: ecological momentary assessment
e-wrapper: engagement wrapper
JITAI: just-in-time adaptive intervention
mHealth: mobile health
MRT: microrandomized trial

## Data Availability

The datasets generated or analyzed during this study will be available from the corresponding author on reasonable request.
